# High Expression of Leucine Zipper-EF-Hand Containing Transmembrane Protein 1 Predicts Poor Prognosis in Head and Neck Squamous Cell Carcinoma

**DOI:** 10.1155/2014/850316

**Published:** 2014-02-05

**Authors:** Liyan Chen, Yang Yang, Shuangping Liu, Longzhen Piao, Yuan Zhang, Zhenhua Lin, Zhuhu Li

**Affiliations:** ^1^Department of Pathology and Cancer Research Center, Yanbian University Medical College, Yanji 133002, China; ^2^Key Laboratory of Natural Resources of Changbai Mountain and Functional Molecules, Yanbian University, Ministry of Education, Yanji 133002, China; ^3^Department of Oncology, Yanbian University Hospital, Yanji 133000, China

## Abstract

Leucine zipper-EF-hand containing transmembrane protein 1 (LETM1) is a mitochondrial inner membrane protein and plays an important role in mitochondrial ATP production and biogenesis. High expression levels of LETM1 have been correlated with numerous human malignancies. This study explored the clinicopathological significance of LETM1 expression as a prognostic determinant in head and neck squamous cell carcinoma (HNSCC). HNSCC samples from 176 patients were selected for immunohistochemical staining of LETM1 protein. Correlations between LETM1 overexpression and clinicopathological features of HNSCC were evaluated by Chi-squared tests and Fisher's exact tests, and relationships between prognostic factors and patient survival were analyzed using Cox proportional hazards models. Our results demonstrated that the strongly positive rate of LETM1 protein was 65.3% in HNSCC, which was significantly higher than in either adjacent nontumor tissue (25.0%) or normal squamous epithelia (6.7%). LETM1 overexpression correlated with poor differentiation, presence of lymph node metastasis, advanced stage, absence of chemoradiotherapy, and 5-year disease-free survival and overall survival rates in HNSCC. Further analysis showed that high LETM1 expression, advanced stage, and nonchemoradiotherapy were significant independent risk factors for mortality in HNSCC. In conclusion, LETM1 plays an important role in the progression of HNSCC and is an independent poor prognostic factor for HNSCC.

## 1. Introduction

Head and neck squamous cell carcinoma (HNSCC) is one of the leading malignancies worldwide [1]. Despite recent advances in treatment modalities, the 5-year survival rate for HNSCC patients has improved only marginally in the past three decades [2, 3]. Metastatic dissemination and tumor recurrence are the major causes of death in HNSCC patients [4, 5]. Therefore, the identification of a reliable biomarker for predicting recurrence and for identifying tumors is of great interest not only for understanding the molecular and cellular processes involved but also for searching for possible new therapeutic molecular targets.

Leucine zipper-EF-hand containing transmembrane protein 1 (LETM1) is one of the mitochondrial inner membrane proteins that is conserved between yeast and humans [6]. The LETM1 gene was first identified as one of the genes deleted in Wolf-Hirschhorn syndrome [7], which is characterized by a contiguous gene disorder resulting from a hemizygous deletion on chromosome 4 [8], and encodes the human homolog of yeast protein Mdm38p. Recent studies have attributed several roles to LETM1, including maintaining mitochondrial morphology, mediating either calcium or potassium/proton antiports, and facilitating mitochondrial translation [9–11]. It is now recognized that most cancer cells predominantly produce energy by glycolysis in the cytoplasm, rather than by oxidative phosphorylation in mitochondria like most normal cells. The importance of this Warburg effect is further underlined by recent studies that report that impaired mitochondrial function renders cancer cells resistant to apoptosis and chemotherapy [12–14]. Additionally, recent studies report that LETM1 may function in mitochondrial biogenesis, which is an important feature of human cancer. Piao et al. reported that LETM1 induced necrotic cell death in HeLa cervical cancer cells by inhibiting mitochondrial biogenesis and mitochondrial ATP production [15]. Hwang et al. reported that adenovirus-mediated overexpression of LETM1 could induce destruction of mitochondria of lung cancer cells through depleting ATP and AMPK activation [16]. However, the role of LETM1 protein in prognostic evaluation and its relationship with survival in HNSCC remain unknown.

To determine whether LETM1 is important in tumorigenesis and to demonstrate its prognostic value in HNSCC, 176 HNSCC samples, 72 adjacent nontumor samples, and 45 normal squamous epithelia samples were selected for immunohistochemical staining of the LETM1 protein. Our data suggest that LETM1 is frequently upregulated in HNSCC compared with either adjacent nontumor tissue or normal squamous epithelia. LETM1 overexpression significantly correlated with poor differentiation, advanced tumor stage, presence of lymph node metastasis, absence of chemoradiotherapy, and shortened survival time in patients with HNSCC. Importantly, the results suggest that LETM1 might be an independent predictor of prognosis in HNSCC.

## 2. Materials and Methods

### 2.1. Ethical Considerations

This research was in accordance with the principles of the Declaration of Helsinki and was approved by the Human Ethics Committee and the Research Ethics Committee of Yanbian University Medical College. Through surgery consent forms, patients were informed that resected specimens were stored by the hospital and potentially used for scientific research, and that their privacy would be maintained. Follow-up survival data were collected retrospectively through medical-record analyses.

### 2.2. Human HNSCC Samples

A total of 293 human tissue samples, including 176 HNSCC samples, 72 adjacent nontumor samples, and 45 normal squamous epithelia samples, were collected from Xi'an Alenabio Company, Affiliated Hospital of Chengde Medical College, and Department of Pathology, Yanbian University Medical College.

The pathological parameters that included gender, age, primary site, tumor differentiation, primary tumor (pT), lymph node, tumor stage, alcohol use, smoking status, and chemoradiotherapy were carefully reviewed in all 176 HNSCC cases. The 176 cases comprised 113 men and 63 women. Patient age (≤60 to >60 years) was 106 : 70. pT sites were accepted tongue (92), buccal (37), gingiva (28), and floor of mouth (19). According to the pT classification, 72 patients had T1-2 tumors and 104 patients had T3-4 tumors. The 176 HNSCC samples were 53 cases of early stage (stages I-II) and 123 cases of advanced stage (stages III-IV) according to *Union for International Cancer Control Criteria* (7th edition) and WHO classification of tumors (*Pathology and Genetics of Head and Neck Tumors*). Fifty-nine patients had well differentiated squamous cell carcinoma, 81 cases were moderately differentiated, and 36 cases were poorly differentiated. All 176 HNSCC patients had follow-up records of more than 5 years, and the follow-up deadline was April 2012. Survival time was counted as the date of surgery to the follow-up deadline or date of death (usually the result of cancer recurrence or metastasis).

### 2.3. Immunohistochemistry for LETM1 Protein in Paraffin-Embedded Tissues

Immunohistochemical staining was performed using the standard streptavidin-peroxidase method. Briefly, sections were deparaffinized, rehydrated, and incubated with 3% H_2_O_2_ in methanol at room temperature for 15 min to deactivate endogenous peroxidase. Antigens were retrieved at 95°C for 20 min by placing sections in 0.01 M sodium citrate buffer (pH 6.0). Sections were incubated with primary antibodies for LETM1 (1 : 50, Catalogue no. H00003954-M03, Abnova, Taipei, Taiwan) at 4°C overnight and washed with phosphate-buffered saline (PBS). After incubation with biotinylated secondary antibodies at room temperature for 30 min, sections were covered with streptavidin-peroxidase complex at room temperature for 30 min. Immunostaining was developed using 3,3'diaminobenzidine chromogen system and counterstained with Mayer's hematoxylin. Mouse IgG isotope was used as the control, with a negative result. The negative control of positive tissue sections was set with PBS instead of the primary antibody.

### 2.4. Evaluation of Immunohistochemical Staining

All sections were evaluated independently by two pathologists without knowledge of the clinical outcome. Interpretation criteria were described previously by Elzagheid et al. and Lin et al. [17, 18]. Briefly, immunostaining for LETM1 was semiquantitatively scored as “−" (no or less than 5% positive cells), “+” (5–25% positive cells), “++” (26–50% positive cells), and “+++” (more than 50% positive cells). For statistical analysis, only cytoplasmic and membranous staining patterns were considered as positive staining. A strongly positive descriptor (LETM1 overexpression) was assigned to “++” and “+++” scored cells. For survival analysis, the LETM1 expression level was denoted as high expression (“++” and “++”) and low expression (“−” and “+”).

### 2.5. Statistical Analysis

Statistical analysis was conducted using SPSS 17.0 software (SPSS Inc., Chicago, IL, USA). Relationships between LETM1 protein expression and clinicopathological parameters were analyzed by Chi-squared tests. Actuarial calculations of disease-free survival and overall survival were obtained using the Kaplan-Meier method. The Cox proportional hazards regression model was used for univariate and multivariate survival analysis. A *P* value of <0.05 was considered statistically significant.

## 3. Results

### 3.1. Expression of LETM1 Protein in HNSCC and Normal Squamous Epithelia

**Figure 1:**
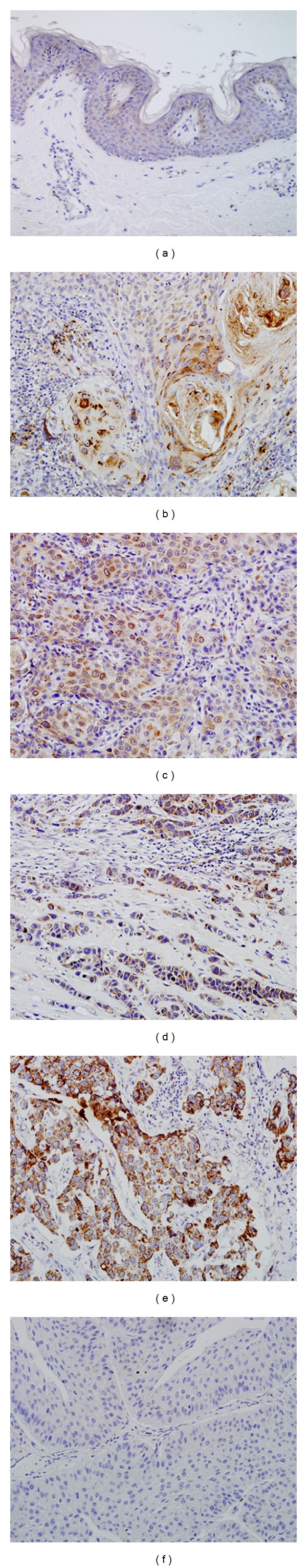
Immunohistochemical staining for LETM1 protein in HNSCC and normal squamous epithelia. (a) LETM1 protein is negative in normal squamous epithelial cells. ((b)–(d)) LETM1 protein showed strongly cytoplasmic positive signals in HNSCC with well (b), moderate (c), and poor (d) differentiation. (e) LETM1 protein is strongly positive in HNSCC with lymph node metastasis. (f) LETM1 protein is negative in HNSCC with no metastasis. (Original magnification, (a)–(f), ×200).

LETM1 protein showed mainly cytoplasmic staining patterns in HNSCC (Figure 1). The positive rate of LETM1 protein expression was 80.7% (142/176) in HNSCC and was significantly higher than that in either adjacent nontumor tissue (51.4%, 37/72) and normal squamous epithelia (22.2%, 10/45) (*P* < 0.001). Similarly, the strongly positive rate of LETM1 protein was 65.3% (115/176) in HNSCC, which was also significantly higher than that in either adjacent nontumor tissues (25.0%, 18/72) or normal squamous epithelia (6.7%, 3/45) (*P* < 0.001) (Figure 1 and Table 1).

### 3.2. Clinicopathological Significance of LETM1 Protein Overexpression in HNSCC

To evaluate the role of LETM1 protein in HNSCC progression, relationships between LETM1 protein expression and the clinicopathological features of HNSCC were analyzed. We found that the strongly positive rate of LETM1 protein was significantly higher in poorly differentiated HNSCC (80.6%, 29/36) than in well (55.9%, 33/59) and moderately (65.4%, 53/81) differentiated cases (*P* = 0.016). Similarly, we found that the strongly positive rate of LETM1 protein was higher in HNSCC with lymph node metastasis (72.2%, 96/133) than in cases with nonmetastasis (44.2%, 19/43) (*P* = 0.001). For tumor stages, in 123 patients with advanced stage HNSCC, 96 (78.0%) had high levels of LETM1 expression, whereas, in the 53 patients with early stage HNSCC, only 19 (35.8%) showed high levels of LETM1 protein expression (*P* < 0.001). Additionally, HNSCC patients who received chemoradiotherapy had lower LETM1 expression (57.3%, 67/117) compared with those who had not received chemoradiotherapy (81.4%, 48/59) (*P* = 0.002). LETM1 expression levels were not related to gender (*P* = 0.545), age (*P* = 0.466), primary site of tumor (*P* = 0.764), pT (*P* = 0.334), alcohol use history (*P* = 0.168), and smoking status of patients with HNSCC (*P* = 0.328) (Table 2).

### 3.3. LETM1 Protein Is an Independent Prognostic Biomarker for HNSCC Using Cox Proportional Hazards Regression Model Analysis

**Figure 2:**
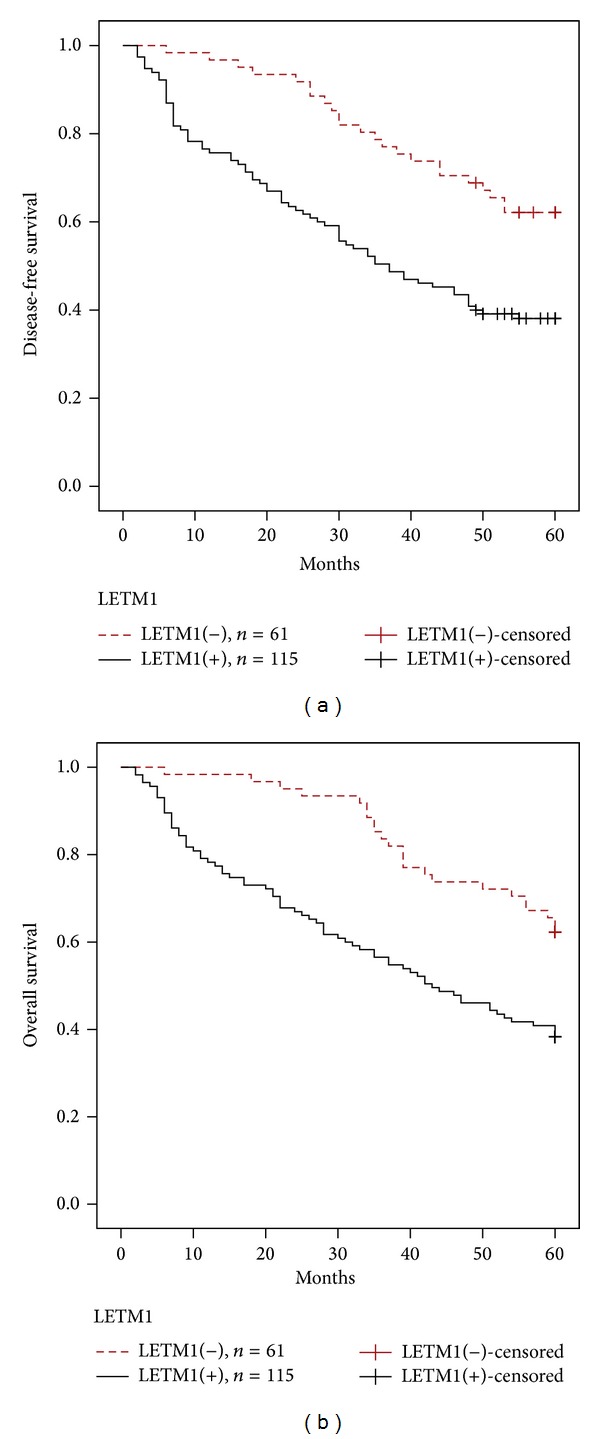
Kaplan-Meier survival curves illustrating the significance of LETM1 expression in HNSCC. HNSCC patients with high LETM1 expression had lower 5-year disease-free survival rates (a) and overall survival rates (b) compared with HNSCC patients with low LETM1 expression, respectively. Log-rank *P* = 0.000 and *P* = 0.000.

**Figure 3:**
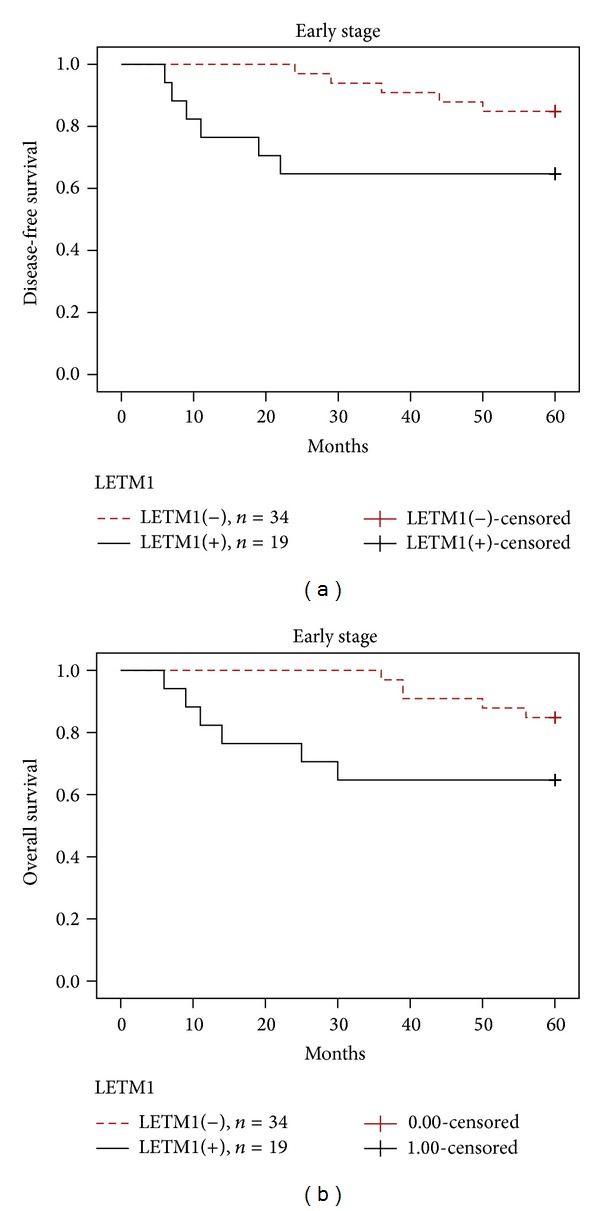
Kaplan-Meier survival curves illustrating the significance of LETM1 expression in early stage HNSCC. In early stage HNSCC (stages I-II, *n* = 53), patients with high LETM1 expression had significantly reduced 5-year disease-free survival rates (a) and overall survival rates (b) compared with those with low LETM1 expression, respectively. Log-rank *P* = 0.046 and *P* = 0.044.

To further substantiate the importance of high LETM1 expression in HNSCC progression, we analyzed disease-free survival and overall survival of 176 HNSCC cases using the Kaplan–Meier method and found that patients with high LETM1 expression had lower 5-year disease-free survival and overall survival than those with low LETM1 expression (*P* < 0.0001) (Figures 2(a)-2(b)). For patients with early stage HNSCC, high levels of LETM1 expression showed worse survival rates than those with low levels of LETM1 expression (*P* = 0.046) (Figures 3(a)-3(b)). LETM1 expression levels were not related to the survival rates of patients with advanced stage HNSCC (data not shown, *P* = 0.374).

Univariate analysis demonstrated that tumor differentiation (*P* = 0.005), TNM stage (*P* = 0.002), chemoradiotherapy (*P* = 0.019), and LETM1 expression status were significantly associated with 5-year disease-free survival and overall survival in patients with HNSCC (Table 3). These data suggest that LETM1 could be a valuable prognostic factor in HNSCC. Therefore, multivariate analysis was performed using the Cox proportional hazards model for all the significant variables examined in the univariate analysis. We found that advanced tumor stage (*P* = 0.033) and treatment with chemoradiotherapy (*P* = 0.001) were independent risk factors. Importantly, LETM1 overexpression also emerged as a significant independent prognostic factor for survival in HNSCC (*P* = 0.033) (Table 4).

## 4. Discussion

The majority of patients with HNSCC are generally diagnosed at an advanced stage, and hence the survival of HNSCC patients is poor [19]. Treatment of HNSCC has evolved over the last two decades to incorporate modalities that have resulted in decreased patient morbidity but with limited success [20]. Therefore, molecular markers for use as prognostic indicators have been studied to improve prediction of the clinical outcome of HNSCC.

LETM1, which was initially discovered as an excellent candidate gene for Wolf-Hirschhorn syndrome, encodes a member of the EF-hand family of Ca(2+)-binding proteins, which contains two EF hands, a transmembrane domain, a leucine zipper, and several coiled-coil domains [7]. On the basis of its possible Ca(2+)-binding property, LETM1 was found to be involved in Ca(2+) signaling and/or homeostasis. LETM1 is an inner mitochondrial membrane protein evolutionarily conserved throughout the eukaryotic kingdom and exhibits homology to Mdm38p, a putative yeast protein involved in mitochondrial morphology. Recent evidence suggests that the LETM1 protein family functions as a key element of mitochondrial volume homeostasis and is also involved in respiratory chain biogenesis. Tamai et al. reported that LETM1 has a distinct role in mitochondrial biogenesis by maintaining mitochondrial volume and shape that helps achieve efficient assembly of respiratory chains [9]. Accumulating evidence suggests that mitochondrial function and cellular metabolism play essential roles in cancer progression and therapeutic resistance [21, 22]. Piao et al. reported that expression levels of LETM1 markedly increased in various human cancers compared with normal tissue counterparts taken from the breast, colon, esophagus, lung, ovary, rectum, stomach, and uterine cervix by immunohistochemistry. Western blot data from liver and colon cancer tissue also demonstrated similar results, showing that high LETM1 protein expression raises the exciting possibility of using LETM1 as a tumor marker [15]. Shin et al. reported that through simultaneous activation of LETM1 and carboxyl-terminal modulator protein, a synergistic antitumor effect was induced in livers of HCC model mice through the downregulation of the Akt1 pathway [23]. Overexpression of LEMT1 was also reported to induce apoptosis in lung cancer cells, which was matched by decreased mitochondrial biogenesis [16]. However, LETM1's function in tumorigenesis and the regulation of its expression levels are largely unclear, and the role of LETM1 as a prognostic biomarker in cancer has not been reported to date.

The overall goal of this study was to determine whether overexpression of LETM1 protein might serve as a biomarker for prognostic evaluation of HNSCC. This is the first study, to the best of the authors' knowledge, to correlate LETM1 expression levels with histological prognostic factors and survival of patients. We performed immunohistochemical staining of LETM1 protein and survival data analysis and found that the positive rate of LETM1 protein was significantly higher in HNSCC tissue (80.7%) than in either adjacent nontumor tissue (51.4%) or normal squamous epithelial tissue (22.2%), indicating that LETM1 protein potentially plays an important role in HNSCC development.

Despite the strong association between LETM1 expression and cancer, there have been no reports of LETM1 expression-based outcomes in tumor patients. In the present study, we found that the strongly positive rate of LETM1 protein was significantly higher in poorly differentiated HNSCC than in well and moderately differentiated cases (*P* = 0.016), and it was also higher in HNSCC with lymph node metastasis than in cases with nonmetastasis (*P* = 0.001). LETM1 overexpression showed a correlation with the TNM stage of HNSCC, which was higher in advanced stage HNSCC (stages III-IV) than in early stage cases (stages I-II) (*P* < 0.001). In addition, the strongly positive rate of LETM1 protein was higher in cases of HNSCC with chemoradiotherapy than in cases without chemoradiotherapy. However, LETM1 expression levels were not correlated with gender, age, pT, alcohol use history, or smoking status of HNSCC patients. These findings raise the possibility that LETM1 facilitates aggressive cancer behavior, resulting in a poor prognosis for patients with HNSCC.

LETM1 overexpression correlated with shortened disease-free survival and overall survival of patients with HNSCC. Additionally, LETM1 overexpression was helpful for predicting the poor survival of patients with early stages of HNSCC (*P* < 0.05). LETM1 expression was not related to survival of patients with advanced stage HNSCC. The high proportion and prognostic value of LETM1 expression in HNSCC suggest that LETM1 may be a potential biomarker for HNSCC. However, more extensive research is needed to clarify the exact roles of LETM1 in the development and progression of HNSCC.

## 5. Conclusions

In this study, we have demonstrated that LETM1 expression may be one of the independent prognostic factors, along with tumor stage and chemoradiotherapy. Taken together, our findings demonstrate that LETM1 plays an important role in the progression of HNSCC, and high levels of LETM1 protein are significantly associated with the presence of lymph node metastasis, advanced stage, poor differentiation, and shortened survival of patients with HNSCC. LETM1 may prove useful as a novel independent biomarker for prognostic evaluation of HNSCC.

## Figures and Tables

**Table 1:** LETM1 protein expression in HNSCC and normal squamous epithelia.

Diagnosis	Number of cases	LETM1 expression				Positive rate (%)	Strongly positive rate (%)
		−	+	++	+++		
SCC	176	34	27	73	42	80.7%**	65.3%**
Adjacent non-tumor	72	35	19	10	8	51.4%**	25.0%*
Normal epithelia	45	35	7	3	0	22.2%	6.7%

SCC: squamous cell carcinoma.

Positive rate: percentage of positive cases with +, ++, and +++ staining score. Strongly positive rate: (high-level expression) percentage of positive cases with ++ and +++ staining score.

*P < 0.05 and **P < 0.01 compared with normal epithelia.

**Table 2:** Correlations between LETM1 protein overexpression and clinicopathological features of HNSCC patients.

Clinical features	Number of cases (*n*)	LETM1 strongly positive cases (%)	*χ* ^2^	*P* value
Gender				
Male	113	72 (63.7%)	0.368	0.545
Female	63	43 (68.3%)		
Age (years)				
≤60	106	67 (63.2%)	0.536	0.466
>60	70	48 (68.6%)		
Primary site				
Tongue	92	56 (60.9%)	4.133	0.764
Buccal	37	29 (78.4%)		
Gingiva	28	19 (67.9%)		
Floor of mouth	19	11 (57.9%)		
Tumor differentiation				
Well	59	33 (55.9%)	5.988	0.016*
Moderately	81	53 (65.4%)		
Poorly	36	29 (80.6%)		
pT				
T1-2	72	42 (58.3%)	0.93	0.334
T3-4	104	53 (50.9%)		
Lymph node				
N_0_	43	19 (44.2%)	11.245	0.001**
N_+_	133	96 (72.2%)		
Tumor stage				
I-II	53	19 (35.8%)	29.13	0.001**
III-IV	123	96 (78.0%)		
Alcohol use				
Positive	113	78 (69.0%)	1.89	0.168
Negative	63	37 (58.7%)		
Smoking status				
Current/former	104	71 (76.0%)	0.963	0.328
Never	72	44 (50.0%)		
Chemoradiotherapy				
Yes	117	67 (57.3%)	10.052	0.002**
No	59	48 (81.4%)		

*P < 0.05 and **P < 0.01.

**Table 3:** Univariate survival analyses (Cox regression model) of various factors in patients with HNSCC.

Factors	Disease-free survival	*P* value	Overall survival	*P* value
	Hazard ratio (95% CI)		Hazard ratio (95% CI)	
Gender	1.135 (0.834–1.545)	0.421	1.049 (0.771–1.428)	0.760
Age	1.223 (0.904–1.655)	0.191	1.221 (0.902–1.651)	0.196
Primary site	0.931 (0.806–1.075)	0.328	0.946 (0.820–1.092)	0.449
differentiation	1.412 (1.154–1.726)	0.001**	1.342 (1.095–1.644)	0.005**
pT	1.759 (1.295–2.390)	0.235	1.366 (1.012–1.846)	0.146
Lymph node	1.108 (0.786–1.563)	0.558	1.068 (0.758–1.507)	0.706
Stage	2.211 (1.581–3.092)	0.000**	1.675 (1.205–2.239)	0.002**
Alcohol use	1.314 (0.964–1.791)	0.085	1.135 (0.979–1.821)	0.068
Smoking	1.243 (0.920–1.679)	0.157	1.127 (0.835–1.521)	0.436
Chemoradiotherapy	2.367 (1.713–3.273)	0.000**	1.948 (1.418–2.675)	0.000**
LETM1	1.628 (1.192–2.224)	0.002**	1.478 (1.083–2.017)	0.014*

**Table 4:** Multivariant survival analyses (Cox regression model) of various factors in patients with HNSCC.

Factors	Disease-free survival	*P* value	Overall survival	*P* value
	Hazard ratio (95% CI)		Hazard ratio (95% CI)	
Differentiation	1.183 (0.955–1.464)	0.124	1.169 (0.946–1.446)	1.446
pT	1.195 (0.847–1.687)	0.311	1.050 (0.740–1.490)	0.783
Stage	1.296 (0.892–1.883)	0.174	1.503 (1.034–2.185)	0.004**
Chemoradiotherapy	1.670 (1.199–2.324)	0.002**	1.734 (1.192–2.522)	0.000**
LETM1	1.080 (0.780–1.495)	0.642	1.412 (1.019–1.956)	0.038*
